# Optical coherence tomography angiography analysis of microvascular abnormalities and vessel density in treatment-naïve eyes with diabetic macular edema

**DOI:** 10.1186/s12886-022-02632-3

**Published:** 2022-11-03

**Authors:** Imène Zhioua Braham, Hela Kaouel, Mejdi Boukari, Imen Ammous, Khalil Errais, Ilhem Mili Boussen, Raja Zhioua

**Affiliations:** 1 Department of Ophthalmology, Charles Nicolle University Hospital, 1007 Tunis, Tunisia; 2 Faculty of Medicine of Tunis, University of Tunis-El Manar, Tunis, Tunisia

**Keywords:** Capillary non perfusion, Capillary plexus, Diabetic macular edema, Diabetic retinopathy, Optical coherence tomography angiography, Vascular density

## Abstract

**Background:**

The aim of this study was to evaluate the structural retinal vascular integrity using optical coherence tomography angiography (OCTA) in treatment-naïve eyes with diabetic macular edema (DME) and to compare it with findings in diabetic eyes without DME.

**Methods:**

In this prospective study, 70 eyes with diabetic retinopathy were included (37 eyes with DME and 33 eyes without DME). The medical records, including swept-source optical coherence tomography and 9 × 9 mm swept-source OCTA images were reviewed and compared between DME and non-DME groups. Microaneurysms, intraretinal microvascular abnormalities (IRMA), areas of capillary non perfusion, foveal avascular zone (FAZ), and capillary vascular density (CVD) were analyzed in the superficial capillary plexus (SCP) and the deep capillary plexus (DCP).

**Results:**

Compared to the non-DME eyes, DME eyes had more microaneurysms in the SCP and the DCP (*p* = 0,039 and *p* = 0,024 respectively), more IRMA in the SCP (*p* = 0,005), larger areas of capillary non perfusion in the SCP and the DCP (*p* = 0,026 and *p* = 0,02 respectively) and larger FAZ in both plexuses (*p* = 0,048 in the SCP and *p* = 0,012 in the DCP). The CVD in the DCP was lower in DME eyes compared to non-DME eyes (*p* = 0,007). The severity of DME was significantly correlated to the number of microaneurysms and to the FAZ surface. Central macular thickness was significantly correlated with the number of microaneurysms in the DCP, the surface of capillary non perfusion areas and the FAZ area in both plexuses.

**Conclusions:**

OCTA with a 9 × 9 mm field of view showed that the retinal vascular integrity regarding the number of microaneurysms, the number of IRMA, the surface of capillary non perfusion areas, the FAZ area and the CVD, was significantly more impaired in DME eyes compared to diabetic eyes without DME. The DCP seemed to be more affected in diabetic eyes with and without DME than the SCP.

## Background

Diabetic macular edema (DME), is the leading cause of visual impairment in diabetic patients worldwide [1, 2]. Although its pathogenesis is still unclear, the increased macular thickness in DME seems to be due to leakage from microaneurysms, capillary hyperpermeability, breakdown of the blood-retinal barrier with increased inflammatory cytokines and vascular endothelial growth factors (VEGF) secondary to capillary drop-out [3–5].

Imaging and monitoring of DME is based mainly on fluorescein angiography (FA) and optical coherence tomography (OCT) [4, 5]. FA is able to analyze vascular changes, leakage and retinal ischemia in the superficial retinal capillary plexus using intravenous dye injection. Optical coherence tomography allows to non-invasively visualize DME features such as cystoid spaces, subretinal fluid, hard exsudates and vitreomacular interface abnormalities. In addition, OCT can identify prognostic and treatment-response factors by analyzing retinal structural integrity and macular thickness.

Optical coherence tomography angiography (OCTA), a new non invasive tool, enables for the first time to study both retinal vascular layers (superficial and deep capillary plexuses) and microstructure abnormalities without dye injection. Detection of vascular changes including microaneurysms, capillary non perfusion areas, erosion of foveal avascular zone (FAZ) and neovessels on OCTA in diabetic retinopathy (DR) has been evaluated [6–9]. In diabetic macular edema, some studies have evaluated FAZ area and vessel density on 3 × 3 mm or 6 × 6 mm scan areas on OCTA in DME eyes that either received previous treatment or being treatment naïve [10–12]. However, few data are available on the role of microaneurysms, intraretinal microvascular abnormalities (IRMA) and capillary non perfusion areas detected by OCTA in DME eyes. In fact, some previous studies analyzed number of microaneurysms, vascular density and FAZ area in DME and non-DME diabetic eyes on 3 × 3 or 6 × 6 mm images. But they didn’t evaluate the number of IRMA, the surface of CPN areas in these eyes. Besides, no study has evaluated these features on a 9 × 9 mm field of view on OCTA in DR and DME.

The aim of this study was to evaluate microaneurysms, IRMA, capillary non perfusion areas, foveal avascular zone and capillary vessel density (CVD) on a 9 × 9 mm OCTA in treatment-naïve DME eyes and to compare them to findings in diabetic eyes without DME.

## Methods

### Study population

This prospective observational study was conducted at Charles Nicolle University Hospital (Tunis, Tunisia) between April 2017 and February 2018. The study adhered to the provisions of the Declaration of Helsinki for research involving human subjects. Informed consent was obtained routinely from all examined patients to participate in this research.

Inclusion criteria were diabetic patients diagnosed with any stage of DR. The clinical severity of DR was classified using the International Clinical Diabetic Retinopathy and Diabetic Macular Edema Severity Scale [13]. We included treatment-naïve eyes with DME and age-matched diabetic eyes without DME. DME was defined as the presence of one or more of these three structural changes on OCT: retinal swelling, cystoid macular edema and subretinal fluid with a central subfield grid thickness of 300 µm or greater. The DME was classified in mild, moderate and severe depending on the distance of the exudates and thickening from the center of the fovea [13].

Exclusion criteria were vitreous hemorrhage, evolved cataract, proliferative diabetic retinopathy complicated with fibrovascular proliferation or tractional retinal detachment or neovascular glaucoma, previous intravitreal anti-VEGF or steroids injection, previous macular laser photocoagulation, previous vitrectomy surgery and any other concomitant ocular disease such as full-thickness macular hole, age related macular degeneration, choroidal neovascularization, high myopia, uveitis and retinal vascular occlusions.

Patients diagnosed with DR underwent detailed ophthalmic examination including measurement of Snellen best-corrected visual acuity (BCVA), slit-lamp biomicroscopy, and dilated fundus examination. Color fundus photography, fluorescein angiography (Topcon TRC-50DX; Topcon Medical Systems, Inc., Tokyo, Japan), and swept-source OCT (DRI OCT Triton plus; Topcon, Tokyo, Japan) were also performed for all patients. Central macular thickness (CMT) was measured in the central 1 mm^2^ subfield of the automatic Early Treatment Diabetic Retinopathy Study (ETDRS) grid. The normal macular thickness of this machine is 283.3 ± 16.5 µm.

### Optical coherence tomography angiography

The instrument used for the OCTA images was based on the DRI OCT Triton plus (Topcon). This system has an A-scan rate of 100,000 scans per second using a light source of 1,050 nm. OCTA volumes were acquired over a 9 × 9 mm field of view in about 10 s of total OCT scan time.

The image processing technology was based on the OCTA Ratio Analysis (OCTARA) detection software with a full-spectrum method. This system extracts the signal changes derived from vascular flow using multiple OCT B-scans acquired at the same position.

Angiograms of the superficial and deep capillary plexuses were automatically segmented using preset parameters. The en-face image was segmented with an inner boundary at 5.6 µm beneath the inner limiting membrane and an outer boundary at 12.6 µm beneath the inner plexiform layer to obtain images of the superficial capillary plexus (SCP), and between boundaries at 15.6 and 70.2 µm beneath the inner plexiform layer for images of the deep capillary plexus (DCP). In the case of incorrect segmentation, we manually adjusted the boundary between the SCP and the DCP.

A total of 25 eyes were excluded from the study because of poor quality OCTA images due to media opacity or significant motion artifact resulting from poor patient cooperation or fixation loss.

### Image analysis and statistical analysis

Quantitative analysis of the 9 × 9 mm OCTA images was performed including counting the number of microaneurysms and the number of IRMA, and measuring the surface of capillary non perfusion areas, the foveal avascular zone and the capillary vessel density in each layer. These measurements were independently conducted by two masked examiners (I.Z.B, H.K). The measurements were averaged to obtain a final value.

**Fig. 1:**
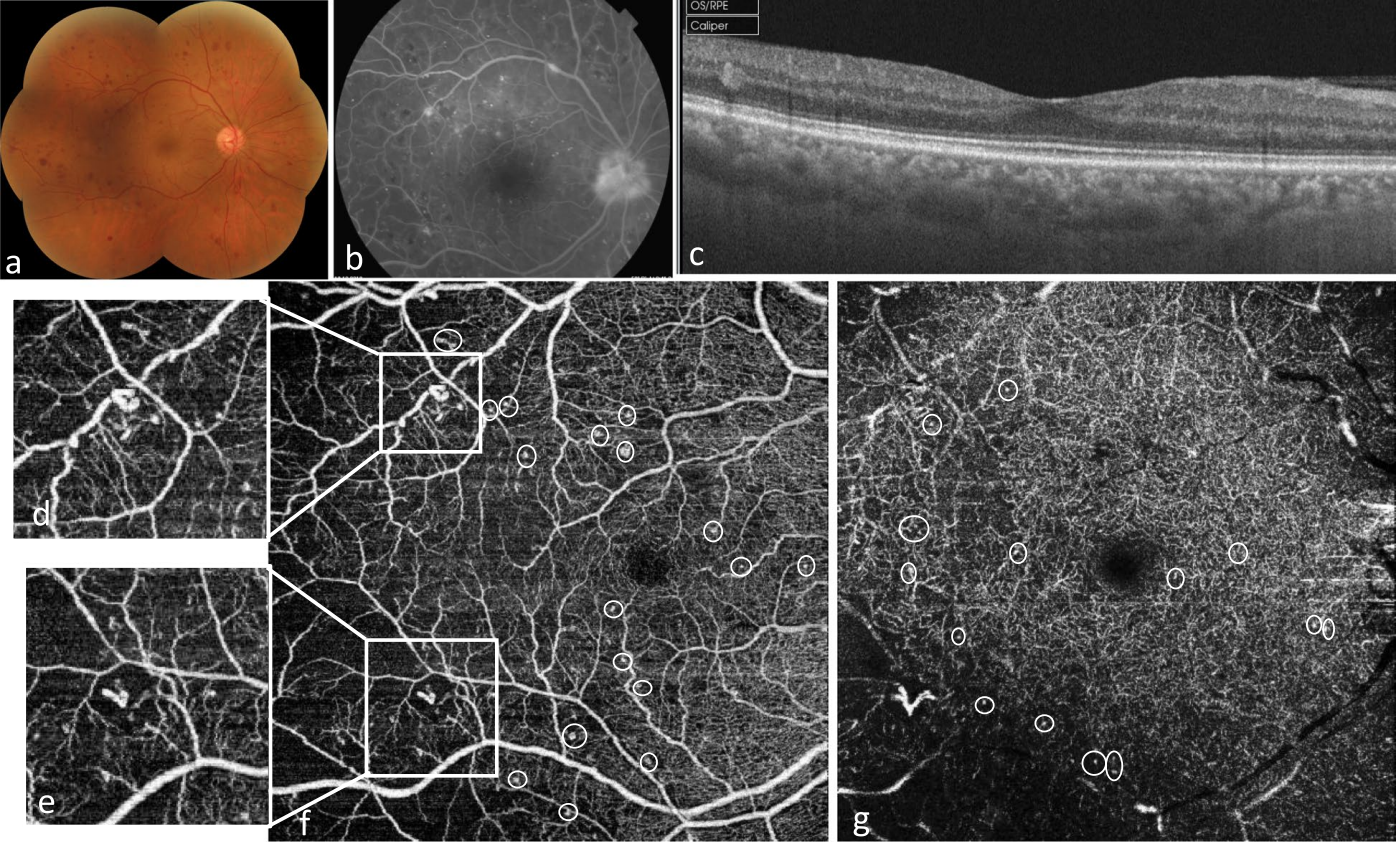
Multimodal imaging of a 54-year-old man with proliferative diabetic retinopathy without diabetic macular edema. **a.** Color fundus photograph shows retinal hemorrhages and papillary neovascularization. **b.** Fluorescein angiography shows multiple microaneurysms, IRMA, papillary neovascularization and areas of peripheral retinal ischemia. **c.** SS-OCT shows absence of macula edema and normal macular structure. **f, d, e, g.** OCTA in the superficial **(f)** and deep capillary plexus **(g)** shows multiple microaneurysms (white circles). IRMA in the superficial capillary plexus are outlined in the two white squares, and magnified in images **d** and **e**

Distinctly round, saccular or fusiform capillary dilation were defined as microaneurysms on OCTA in this study. IRMA were defined as vascular budding located at the edge or within a non-perfusion capillary area. The number of microaneurysms and the number of IRMA in each vascular layer were manually counted (Fig. 1).

**Fig. 2:**
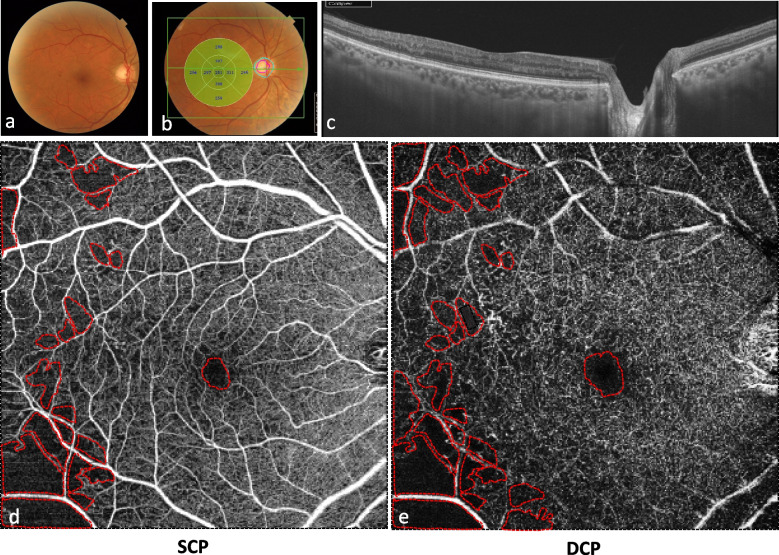
OCTA in a 63-year-old man with severe non proliferative diabetic retinopathy without diabetic macular edema. **a, b.** Color fundus photographs shows microaneurysms and retinal hemorrhages and normal macular thickness. **c**. SS-OCT shows absence of macula edema and normal macular structure. **d, e.** Non perfusion capillary areas (NPCA) and foveal avascular zone (FAZ) were manually delineated in red on the OCTA. The superficial capillary plexus **(d)** showed NPCA of 9.223 mm^2^ and a FAZ area of 0,357 mm^2^. The deep capillary plexus **(e)** showed larger NPCA of 12,98 mm^2^ and a FAZ area of 0,430 mm^2^

**Fig. 3:**
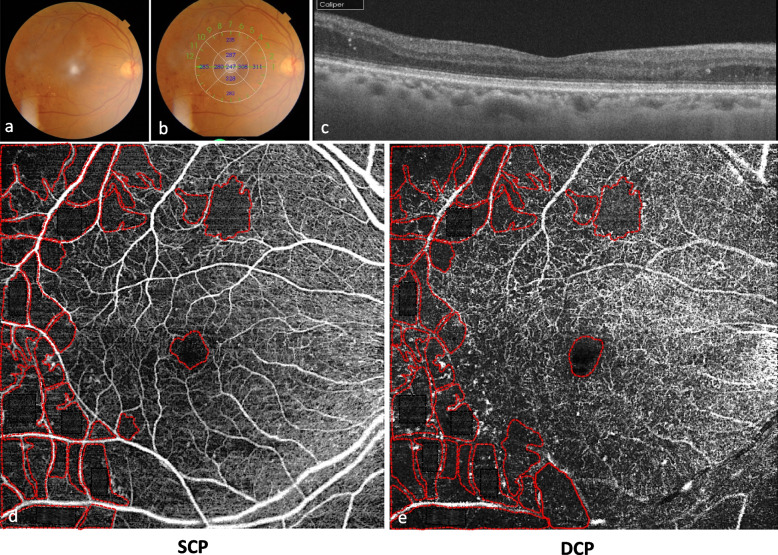
OCTA in a 63-year-old man with severe non proliferative diabetic retinopathy with mild diabetic macular edema. **a, b.** Color fundus photographs shows microaneurysms and retinal hemorrhages and extrafoveal increased macular thickness. **c**. SS-OCT shows mild macula edema with extrafoveal cysts and hard exudates. **d, e.** Non perfusion capillary areas (NPCA) and foveal avascular zone (FAZ) were manually delineated in red on the OCTA. The superficial capillary plexus **(d)** showed NPCA of 23,422 mm^2^ and a FAZ area of 0,424 mm^2^. The deep capillary plexus **(e)** showed larger NPCA of 28,912 mm^2^ and a FAZ area of 0,446 mm^2^

Quantitative assessment of surface of capillary non perfusion areas, FAZ and CVD was performed using ImageJ software (developed by Wayne Rasband, National Institutes of Health, Bethesda, MD; available at: http://rsb.info.nih.gov/ij/index.html). The total surface of all non perfusion areas and the FAZ area were manually marked and measured in square millimeters (Figs. 2 and 3).

The intraclass correlation coefficient was used to determine the interobserver agreement for the manually measured FAZ area.

The original OCTA images of the SCP and the DCP were binarized to convert them from gray scale into black and white images using the ImageJ software. Capillary vessel density defined as the percentage area occupied by the blood vessels, was calculated by counting the number of black pixels and total pixels.

Statistical analyses were performed using SPSS software version 19.0 (SPSS Inc, Chicago, IL). Descriptive statistics of continuous variables were presented by percentages, mean, standard deviation and minimum and maximum of the range. For quantitative data, comparisons between groups were evaluated using the Student t test, Kruskal–Wallis, Mann et Whitney test, or McNemar test, as appropriate. Pearson’s “r” correlation was measured to analyze the degree of correlation between sets of data. Interobserver reproducibility was determined using Cohen’s kappa and the intraclass correlation coefficient. A P value < 0.05 was considered statistically significant.

## Results

This prospective observational study included 37 diabetic eyes with DME, and 33 diabetic eyes without DME of 45 patients at different stages of DR, imaged using 9 × 9 mm OCTA. No significant difference was found between both groups, regarding age, sex and stage of DR. Diabetic eyes with macular edema had significantly thicker retina, than diabetic eyes without edema with a CMT of 388.189 ± 80.179 µm and 246.969 ± 25.238 µm, respectively (*P* < 0,001). A significantly worse visual acuity was found in diabetic eyes with macular edema (mean ± SD LogMAR = 0.6 ± 0.38), compared to diabetic eyes without edema (0.18 ± 0.24; *P* = 0,001), as seen in Table 1.

**Table 1:** Demographic and clinical data of the diabetic macular edema and nondiabetic macular edema eyes

	DME eyes (*N* = 37)	Non-DME eyes (*N* = 33)	*P* value
Age			
Range	(46–80)	(20–70)	0,097
Mean ± SD	59 ± 10,25	57,70 ± 13,26	
Sex			
Male (*n* = %)	20 (54)	15 (45)	0,472
Female (*n* = %)	17 (45)	18 (54)	
BCVA (LogMar)			
Range	(0,1- 1,3)	(0–1)	**0,001**
Mean ± SD	0,605 ± 0,383	0,18 ± 0,247	
CMT (µm)			
Range	(300–697)	(185–287)	** < 0,001**
Mean ± SD	388,18 ± 80,17	246,96 ± 25,23	
DR stage (n)			
Mild NPDR	2	4	0,746
Moderate NPDR	15	14	
Severe NPDR	10	8	
PDR	10	7	

*DME* diabetic macular edema, *SD* standard deviation, *BCVA* best-corrected visual acuity, *LogMar* logarithm of the minimum angle resolution, *CMT* central macular thickness, *DR* diabetic retinopathy, *NPDR* non proliferative diabetic retinopathy, *PDR* proliferative diabetic retinopathy

The comparison of OCTA findings between DME eyes and non-DME eyes is summarized in Table 2. Diabetic eyes with macular edema had significantly higher number of microaneurysms at the level of the superficial and the deep capillary plexuses (*P* = 0,039 and *P* = 0,024, respectively) than diabetic eyes without edema. Compared to non-DME eyes, DME eyes showed significantly higher number of IRMA in the SCP (*P* = 0,005).

**Table 2:** Optical coherence tomography angiography parameters in 9 × 9 mm scans, centered on the macula, in diabetic eyes with and without diabetic macular edema

	DME eyes (*N* = 37)	Non-DME eyes (*N* = 33)	*P* value
SCP			
Microaneurysms (mean, n ± SD)	25,75 ± 15,94	19,21 ± 14,57	**0,039**
IRMA (mean, n ± SD)	4,08 ± 4,83	1,66 ± 2,64	**0,005**
Capillary non perfusion areas (mean, mm^2^ ± SD)	6,128 ± 9,664	2,441 ± 4,498	**0,026**
FAZ area (mean, mm^2^ ± SD)	0,468 ± 0,415	0,390 ± 0,286	**0,048**
Vascular density (mean, % ± SD)	29,40 ± 8,10	30,75 ± 10,69	0,409
DCP			
Microaneurysms (mean, n ± SD)	48,40 ± 32,51	35,03 ± 21,31	**0,024**
IRMA (mean, n ± SD)	4,11 ± 4,68	3,06 ± 4,24	0,168
Capillary non perfusion areas (mean, mm^2^ ± SD)	7,33 ± 10,735	2,876 ± 4,913	**0,02**
FAZ area (mean, mm^2^ ± SD)	0,720 ± 0,576	0,485 ± 0,383	**0,012**
Vascular density (mean, % ± SD)	29,22 ± 6,86	31,35 ± 8,71	**0,007**

*DME* diabetic macular edema, *SCP* superficial capillary plexus, *SD* standard deviation, *IRMA* intraretinal microvascular abnormalities, *FAZ* foveal avascular zone, *DCP* deep capillary plexus

A significantly larger surface of non perfusion capillary areas was found in the DME eyes group in both superficial and deep capillary plexuses (*P* = 0,026 and *P* = 0,02, respectively) compared to the non-DME eyes group.

Diabetic eyes with macular edema had significantly larger FAZ area than diabetic eyes without edema at the level of the superficial (mean FAZ ± SD = 0,720 ± 0,576 and 0,468 ± 0,415; respectively, *P* = 0,048) and the deep capillary plexuses (mean FAZ ± SD = 0,485 ± 0,383 and 0,390 ± 0,286; respectively, *P* = 0,012).

DME eyes had significantly lower CVD values in the DCP than non-DME eyes (*P* = 0,007). However, the difference of the CVD in the SCP between the DME and non-DME eyes did not quite reach the level of significance.

Table 3 compares the findings in the superficial and deep capillary plexuses on OCTA. The DCP was more affected in both DME eyes and non-DME eyes with a statistically higher number of microaneuryms and a larger FAZ area than in the SCP. The non-DME eyes group showed a statistically higher number of IRMA in the DCP. However, no significant difference in capillary non perfusion areas and vascular density was found between the SCP and the DCP in both DME and non-DME eyes.

**Table 3:** Comparisons of optical coherence tomography angiography findings in 9 × 9 mm scans, centered on the macula, between the superficial capillary plexus and the deep capillary plexus, in diabetic eyes with and without diabetic macular edema

	DME eyes (*N* = 37)			Non-DME eyes (*N* = 33)			All diabetic eyes (*N* = 70)		
	SCP	DCP	P	SCP	DCP	P	SCP	DCP	P
Microaneurysms (mean, n ± SD)	25,75 ± 15,94	48,40 ± 32,51	** < 0,001**	19,21 ± 14,57	35,03 ± 21,31	** < 0,001**	22,67 ± 15,55	42,1 ± 28,41	** < 0,001**
IRMA (mean, n ± SD)	4,08 ± 4,83	4,11 ± 4,68	0,753	1,66 ± 2,64	3,06 ± 4,24	**0,001**	3 ± 4,06	3,6 ± 4,47	** < 0,001**
Capillary non perfusion areas (mean, mm^2^ ± SD)	6,128 ± 9,664	7,33 ± 10,735	0,143	2,441 ± 4,498	2,876 ± 4,913	0,244	4,558 ± 8,011	5,217 ± 8,709	0,05
FAZ area (mean, mm^2^ ± SD)	0,468 ± 0,415	0,720 ± 0,576	** < 0,001**	0,390 ± 0,286	0,485 ± 0,383	**0,001**	0,432 ± 0,359	0,609 ± 0,505	** < 0,001**
Vascular density (mean, % ± SD)	29,40 ± 8,10	29,22 ± 6,86	0,874	30,75 ± 10,69	31,35 ± 8,71	0,118	30,04 ± 9,36	30,23 ± 7,80	0,77

*DME* diabetic macular edema, *SCP* superficial capillary plexus, *DCP* deep capillary plexus, *SD* standard deviation, *IRMA* intraretinal microvascular abnormalities, *FAZ* foveal avascular zone

In the DME group, the stage of DME was mild in 13 eyes (35,1%), moderate in 11 eyes (29,7%) and severe in 13 eyes (35,1%). The severity of DME was statistically correlated to the number of microaneurysms in the SCP (*P* = 0,049). When comparing mild and moderate DME eyes to severe DME eyes, a significant difference was also found in the number of microaneurysms in the DCP (*P* = 0,038). The FAZ area in the DCP was also correlated to the severity of the DME (*P* = 0,009). However, no significant correlation between severity of DME with capillary non perfusion areas and vascular density was found.

Central macular thickness was significantly correlated with the number of microaneurysms in the DCP and with the surface of capillary non perfusion areas and the FAZ area in both plexuses. The CMT was also negatively correlated to BCVA. These correlations are summarized in Table 4.

**Table 4:** Correlation between central macular thickness and microaneurysms, irma, capillary non perfusion areas, faz area and cvd (at the level of the superficial and deep retinal plexuses)

*N* = 70 eyes	Central Macular Thickness	
	R	*P* value
Microaneurysms		
SCP	0,195	0,06
DCP	0,307	**0,006**
IRMA		
SCP	0,176	0,08
DCP	0,055	0,33
Capillary non perfusion areas		
SCP	0,321	**0,04**
DCP	0,245	**0,036**
FAZ area		
SCP	0,299	**0,008**
DCP	0,492	** < 0,0001**
Vascular density		
SCP	-0,042	0,37
DCP	-0,030	0,407
BCVA	-0,435	**0,006**

*SCP* superficial capillary plexus, *DCP* deep capillary plexus, *IRMA* intraretinal microvascular abnormalities, *FAZ* foveal avascular zone, *CVD* capillary vascular density, *BCVA* best-corrected visual acuity

The interobserver agreement was satisfactory for measurement of FAZ area in both the superficial (intraclass correlation coefficient 0,89) and the deep capillary plexus (intraclass correlation coefficient 0,67).

## Discussion

Several studies using OCTA have shown that diabetic eyes (with or without DME) had larger FAZ area and impaired CVD compared to control healthy eyes.

In the present study, we investigated retinal microvascular abnormalities and vessel density on OCTA with a 9 × 9 mm scanning area in treatment-naïve diabetic eyes with DME. To our knowledge, this is the first study using a 9 × 9 mm field of view on OCTA in diabetic eyes (with and without DME). This field of view was useful to detect retinal abnormalities beyond the 6 × 6 mm field in order to have a more accurate analysis of the posterior pole. DME eyes showed significantly higher number of microaneurysms, higher number of IRMA, larger FAZ area and larger surface of capillary non perfusion areas on OCTA than non-DME eyes. The CVD was significantly lower in DME eyes compared to non-DME eyes in the DCP.

Optical coherence tomography angiography allowed to identify and manually count microaneurysms in both retinal layers, as reported by Ishibazawa et al. [6], Jia et al. [7] and Couturier et al. [8]. In this study, the number of microaneurysms was higher in DME eyes compared to non-DME eyes in the SCP and the DCP. Lee et al. calculated the number of microaneurysms on OCTA with a 3 × 3 mm scanning area in DME eyes treated with intravitreal anti-VEGF injections, and found that it was higher than in non-DME eyes in both plexuses [12]. Besides, our results concluded that the number of microaneurysms was correlated with the severity of DME in both plexuses and with the CMT in the DCP. To our knowledge, this is the first study to evaluate the association between microaneurysms and the severity of DME. Hasegawa et al. calculated the density of microaneurysms (6 × 6 mm protocol) and found that it correlated significantly with the macular volume in the DCP [14]. These results confirm that microaneurysms of the DCP play a major role in the pathogenesis of DME.

This current study showed a significantly higher number of IRMA in DME eyes compared to the non-DME eyes in the SCP but not in the DCP. This may be explained by the reduced visualization of vascular abnormalities in the DCP due to the presence of intra-retinal cysts. In another hand, previous studies showed that IRMA were associated with capillary non perfusion in DR with and without DME. This suggests that more retinal ischemia will be associated with more edema and more IRMA [9, 10, 15, 16].

On OCTA, non perfusion areas appear as black areas of flow void delimited by interrupted capillaries. They must be differentiated from macular cysts which are elongated or round shaped and well-delimited black areas [15]. Mané et al. found that in chronic DME, cystoid spaces were located within capillary dropout areas [16]. Spaide also suggested that there is decreased or absent flow signal in the DCP in the region of the cystoid spaces and that reperfusion is partial after resolution of the edema after treatment [17]. However, only few previous studies performed a quantitative assessment of capillary non perfusion areas on OCTA in DR. Ishibazawa et al. quantitatively analyzed the non-perfused areas around the macula in seven diabetic eyes on OCTA and found that these areas were significantly larger in the SCP than in the DCP [6]. Nesper et al. measured the percent area of non perfusion and found that it increased significantly with increasing severity of DR in both plexuses [18]. However, to the best of our knowledge, this is the first study to evaluate the surface of non perfusion capillary areas on OCTA in DME. In our study, we found that the total surface of capillary non perfusion areas was higher in eyes with DME than in eyes without DME in both plexuses. Our results support the hypothesis that DME develops preferably in ischemic areas which is consistent with the pathogenesis of DME.

FAZ area in diabetic eyes with and without DME has been analyzed in some studies. Lee et al. found that DME eyes had larger FAZ area on OCTA (measured on 3 × 3 mm images) than non-DME eyes only in the DCP; and showed that poor responders to anti-VEGF treatment had a larger FAZ area in the DCP than good responders [12]. However, AttaAllah et al. found a significantly larger FAZ area in DME eyes compared to non-DME eyes in the SCP but not in the DCP [19]. The present study showed that the FAZ area was significantly larger in the DME group than in the non-DME group at the level of both capillary plexuses. Although these previous studies used a 3 × 3 mm or 6 × 6 mm scanning areas, their findings were compatible with our current results.

Capillary vessel density is an objective tool on OCTA to evaluate areas of non-perfused retina in order to improve monitoring and management of DR. Several studies using OCTA showed decreased CVD in diabetic eyes compared to healthy eyes. Mané et al. found that in patients with chronic DME, the capillary density was lower than in healthy subjects [16]. AttaAllah et al. also showed a decreased macular vessel density in treatment-naïve DME eyes when compared to controls, however no difference was observed between DME group and non-DME group regarding whole image vessel density at either the SCP or the DCP [19]. In our study we found that eyes with DME exhibited significantly lower CVD values on 9 × 9 mm scanning areas only in the DCP, confirming the findings of the report by Lee et al. on 3 × 3 mm OCTA images [12]. Our results suggest that the decrease of CVD on OCTA, not only in the parafoveal area but also in the posterior pole, may be related to the occurrence of DME. This imply that DME affects more the perfusion of the deep retinal layers than the superficial retinal layers.

The important role of the DCP has been demonstrated in many retinal diseases since the use of OCTA. In DR, the DCP was shown to be affected more than the SCP. In fact, several studies confirmed that vascular anomalies are mostly found in the deep vascular layers, where diabetic vascular changes primarily occur [14]. However, the functional roles of the DCP and their relationship with DME are still unclear. In this study, we demonstrated that the DCP was more affected in both DME eyes and non-DME eyes with a statistically higher number of MA and a larger FAZ area than in the SCP. Nevertheless, we found that CVD was lower in DME eyes compared to diabetic eyes without DME at the level the DCP. We think that disorganisation and non perfusion of the DCP play a major role in the occurrence of DME. This may be explained by the fact that the DCP participates with the choriocapillaris in the oxygen supply of the external layers of the retina [20]. Spaide also hypothesized that the DCP seems to be more at risk for flow problems induced by an increased VEGF in diabetic eyes [17].

Our study has some limitations. The study population included diabetic patients with different DR stages, having or not DME, with or without previous PPR; which can be confounding factors for estimating visual function. Nevertheless, to the best of our knowledge, this is the first study using OCTA with a 9 × 9 mm protocol in DR and to study the surface of capillary non perfusion areas and the number of IRMA in DME. Besides, only very-high quality of images was selected, although 9 × 9 mm scanning areas need very good fixation of patients to avoid motion artefact. It must be also noted that assessment of FAZ area demonstrated a high level of interobserver agreement. Further studies are required to define accurate quantitative methods in monitoring DME on OCTA.

In conclusion, OCTA with a 9 × 9 mm field of view showed that the retinal vascular integrity was more impaired in DME eyes compared to diabetic non-DME eyes. These vascular changes concerned the number of microaneurysms, the number of IRMA, the surface of capillary non perfusion areas, the FAZ area and the CVD. The DCP seemed to be more affected in diabetic eyes with and without DME than the SCP. Our present findings could be useful to establish further objective and quantitative criteria for management and monitoring DME.

## Data Availability

The datasets generated and analyzed during the current study are not publicly available due to patient privacy and intellectual property protection, but are available from the corresponding author on reasonable request.

## Abbreviations

DME: Diabetic macular edema
VEGF: Vascular endothelial growth factor
FA: Fluorescein angiography
OCT: Optical coherence tomography
OCTA: Optical coherence tomography angiography
FAZ: Foveal avascular zone
DR: Diabetic retinopathy
IRMA: Intraretinal microvascular abnormalities
CVD: Capillary vessel density
PRP: Panretinal photocoagulation
BCVA: Best-corrected visual acuity
CMT: Central macular thickness
ETDRS: Automatic Early Treatment Diabetic Retinopathy Study
OCTARA: OCTA Ratio Analysis
SCP: Superficial capillary plexus
DCP: Deep capillary plexus
